# Reply to Geoffrion, D.; Harissi-Dagher, M. Comment on “Wróblewska-Czajka et al. Outcomes of Boston Keratoprosthesis Type I Implantation in Poland: A Retrospective Study on 118 Patients. *J. Clin. Med.* 2024, *13*, 975”

**DOI:** 10.3390/jcm13216497

**Published:** 2024-10-30

**Authors:** Ewa Wróblewska-Czajka, Dariusz Dobrowolski, Ula V. Jurkunas, Edward Wylęgała, Adam Wylęgała

**Affiliations:** 1 Clinical Department of Ophthalmology, Faculty of Medical Sciences in Zabrze, Medical University of Silesia, 40-760 Katowice, Poland; dardobmd@wp.pl (D.D.); ewylegala@sum.edu (E.W.); 2 Ophthalmology Department, Railway Hospital in Katowice, 40-760 Katowice, Poland; 3 Department of Ophthalmology, Santa Barbara Hospital, 41-200 Sosnowiec, Poland; 4 Cornea Center of Excellence, Schepens Eye Research Institute, Massachusetts Eye and Ear Infirmary, Department of Ophthalmology, Harvard Medical School, Boston, MA 02114, USA; ula_jurkunas@meei.harvard.edu; 5 Experimental Ophthalmology Unit, Department of Biophysics, II School of Medicine with the Division of Dentistry in Zabrze Medical University of Silesia, 40-752 Katowice, Poland

Thank you for your thoughtful and insightful commentary on our paper, “Outcomes of Boston Keratoprosthesis Type I Implantation in Poland: A Retrospective Study on 118 Patients” [1]. We are pleased to see your recognition of our study’s contributions to understanding the efficacy and challenges of Boston Keratoprosthesis (BI-KPro) implantation [2] and welcome the opportunity to address the points you raised.

Regarding the incidence of de novo glaucoma in our study, we fully acknowledge its critical impact on the long-term outcomes of BI-KPro implantation, particularly in patients with autoimmune diseases. As noted, we observed a high incidence of glaucoma (66.1%), which corroborates your findings on the increased glaucoma risk in this patient group [3]. Our initial study did not specifically examine the glaucoma rates by subgroup; we did this in our reply (Table 1). Although mucous membrane pemphigoid (MMP) and Stevens–Johnson syndrome (SJS) had the highest increase in glaucoma rate, we did not observe any new glaucoma in the other autoimmune disease group.

We concur that autoimmune patients may be at higher risk [4], particularly given their susceptibility to inflammation and corneal melting [5]. We agree with your recommendation that prompt surgical intervention for glaucoma may optimize visual outcomes in this challenging cohort.

Your focus on the complexity of managing autoimmune conditions aligns with our observation of worse long-term visual acuity and higher complication rates in patients with conditions such as MMP. The increased incidence of corneal melt and retroprosthetic membrane (RKM) formation in this group is indeed multifactorial. We emphasize that careful preoperative assessment of inflammatory risk factors is critical, as is the need for ongoing postoperative vigilance.

We also appreciate your recognition of our findings related to the diversity of visual outcomes based on different surgical indications. Our results indicate significant improvement for patients with ocular burns, a subgroup where fewer RKMs were observed. As you noted, a personalized approach to treatment selection is imperative in maximizing outcomes [3].

Once again, we are grateful for your positive reception of our work and for contributing to a deeper discussion of these important topics.

## Figures and Tables

**Table 1:** Comparison of Glaucoma Prevalence Before and After Procedure, Grouped by Indication.

Indication	Glaucoma Before	Glaucoma After	% Increase
GF	25	33	32
OB	17	24	41.18
NK	3	6	100
MMP	2	5	150
OA	3	3	0
SJS	2	4	100
A	2	3	50

GF = graft failure, OB = ocular burn, NK = neurotrophic keratopathy, MMP = mucous membrane pemphigoid, OA = other autoimmune disease, SJS = Stevens–Johnson syndrome, A = aniridia.
